# Benchmarking the HRP-2 Humanoid Robot During Locomotion

**DOI:** 10.3389/frobt.2018.00122

**Published:** 2018-11-08

**Authors:** Olivier Stasse, Kevin Giraud--Esclasse, Edouard Brousse, Maximilien Naveau, Rémi Régnier, Guillaume Avrin, Philippe Souères

**Affiliations:** ^1^Laboratoire d'Analyse et d'Architecture des Systèmes, CNRS, Université de Toulouse, Toulouse, France; ^2^Laboratoire Nationale de Métrologie et d'Essais, Paris, France; ^3^Max-Planck Institute, Tuebingen, Germany

**Keywords:** benchmarking, bipedal locomotion, humanoid robot HRP-2, controlled environment, numerical optimization, walking

## Abstract

In this paper we report results on benchmarking a HRP-2 humanoid robot. The humanoid robots of this serie are known to be very robust. They have been successfully used by several research groups for the design of new motion generation algorithms. As such it is a reference in the category of electrically driven humanoid robot. As new humanoid robots are continuously built it is interesting to compare the performances of these new prototypes to those of HRP-2. This benchmarking study was realized through a campaign of measurements in an advanced equipped testing laboratory that provides a well adapted controlled environment. We have investigated the effect of temperatures variation on the robot walking capabilities. In order to benchmark various environmental conditions and algorithms we computed a set of performance indicators for bipedal locomotion. The scope of the algorithms for motion generation evaluated here ranges from analytical solution to numerical optimization approach, enabling real-time walking or multi-contacts motions.

## 1. Introduction

**Figure 1:**
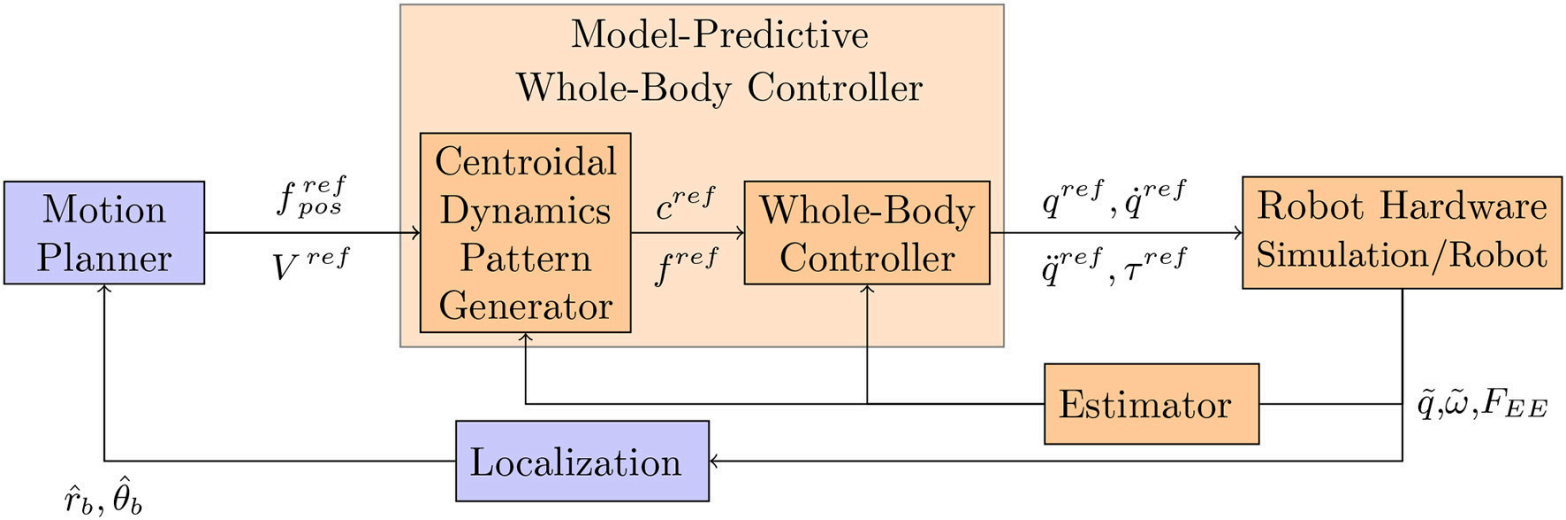
General architecture used to generate humanoid robot motions. In this paper the modules in the orange boxes are the ones that are benchmarked, whereas those in blue are not benchmarked.

From the seminal work of Chestnutt (2010) to the recent methods proposed in the frame of the Darpa Robotics Challenge (DRC) (Radford et al., 2015; DeDonato et al., 2017; Johnson et al., 2017; Lim et al., 2017; Marion et al., 2017; Tsagarakis et al., 2017), humanoid robots use for moving a control architecture that roughly follows the general framework depicted in Figure 1. Based on an internal representation of the environment and the localization of the robot ($\hat{r}b$ and $\hat{\theta }b$ being, respectively, the base position and orientation), the Motion Planner (MP) plans a sequence of reference end-effector contact positions (*f*^*ref*^), or a reference center of mass linear velocity combined with a reference waist angular velocity (*V*^*ref*^). These references are then provided to a Model-Predictive Whole-Body Controller (MPWBC) which generates a motor command for each joint (joint torques (τ^*ref*^), positions (*q*^*ref*^), velocities (${\overset{{}^{\circ}}{q}}^{ref}$) and accelerations (${\ddot{q}}^{ref}$)). This block is critical in terms of safety as it maintains the dynamic feasibility of the control and the balance of the robot. The Model-Predictive Whole-Body Controller can be expressed as a unique optimal control problem but at the cost of efficiency in terms of computation time or solution quality. This is why this controller is usually organized in two stages. First, trajectories for the robot center of mass *c*^*ref*^ and the positions of contacts with the environment *f*^*ref*^ are found using a Centroidal Dynamics Pattern Generator (CDPG). Then, a Whole-Body Controller (WBC) computes an instantaneous controller enabling to track these trajectories. More details about the CDPG can be found in the next paragraph. The whole body reference is in turn sent to the Robot Hardware, which can be either the simulator or the real robot. The feedback terms are based upon the measurements of the different sensors. The encoders evaluate the joint position ($\tilde{q}$). The inertial measurement unit (IMU) measures the angular velocity (${\tilde{\omega }}_{IMU}$) and the linear acceleration (ã_*IMU*_) of the robot torso, which give information about the orientation of the robot with respect to the gravity field. Finally the interaction with the environment is provided by the force sensors classically located at the end-effectors (*F*_*EE*_∈{*F*_*RF*_, *F*_*LF*_, *F*_*RH*_, *F*_*LH*_}, where the subscripts have the following meaning: (*EE*): end-effector, (*RF*): right foot, (*LF*): left foot, (*RH*): right hand, (*LH*): left hand). All these information are treated in an Estimator to extract the needed values for the different algorithms. Finally the Localization block is used to locate as precisely as possible the robot in its 3D environment. Various implementations of this architecture have been proposed with various levels of success from the highly impressive Boston Dynamics System, to robots widely available such as Nao. An open question is the robustness and the repeatability of such a control system as well as its performance. In this paper our main contribution is to propose a benchmarking of the HRP-2 robot in various setups and provide performance indicators in scenarios which are possibly interesting for industrial applications. We hope this study will provide a quantitative comparison and will serve as a baseline for the elaboration of new algorithms. In addition we believe that this paper is one of the first attempt to apply the detailed performance indicators provided by Torricelli et al. (2015) to a human size humanoid robot. The paper is structured as follows: firstly, the section 2 presents the related work on control and benchmarking for humanoid robots. Then section 2.3 depicts our precedent contribution in the Koroibot project and how it relates to this work. To continue, section 3 lists the materials and different methods used to perform the benchmarking. In turn section 9 shows the experimental results using the indicators from section 3. Finally the conclusion in section 5 summaries the contributions and results of the study.

## 2. Related work

In this paragraph we present the work that has been done relative to the control and the benchmarking of the HRP2 humanoid robot.

### 2.1. Motion generation for humanoid robots

The different benchmarks included in this paper are relative to the MPWBC sketched in Figure 1. This related work is presented in this first subsection. Several techniques are used to mathematically formulate this problem. For instance hybrid-dynamics formulations as proposed by Grizzle et al. (2010) or Westervelt et al. (2007) are efficient but difficult to generalize. The approaches used in this paper are based on mathematical optimization which is broadly used in the humanoid robotics community. More precisely, the locomotion problem can be described as an Optimal Control Problem (OCP). The robot generalized configuration (*q*^*ref*^) and velocity (${\overset{{}^{\circ}}{q}}^{ref}$) usually compose the state (**x**∈ℝ^*n*^). The future contact points can be precomputed by a Motion Planner or included in the state of the problem. The control of this system **u**∈ℝ^*m*^, can be the robot generalized acceleration (${\ddot{q}}^{ref}$), the contact wrench (ϕ_*k*_ with *k*∈{0, …, Number of Contact}), or the motor torques (τ^*ref*^). We denote by **x** and **u** the state and control trajectories. The following optimal control problem (OCP) represents a generic form of the locomotion problem (which can be for instance a direct multiple shooting problem):

(1) $$\underset{\underline{x},\;\underline{u}}{\min}\overset{S}{\underset{s\;=\;1}{\sum }}\int_{t_{s}}^{t_{s}+\Delta t_{s}}\ell_{s}(x,u)dt$$

(2) $$s.t.\;\forall t\dot{x}=dyn(x,u)$$

**Figure 2:**
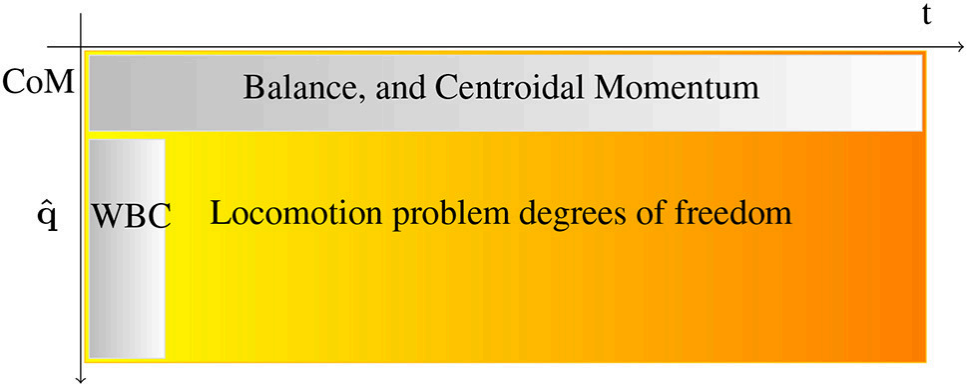
Representation of the dimension of the locomotion problem. The abscissa represents the duration of the predicted horizon and the ordinate the number of robot DoF.

(3) ∀t  ϕ∈K

(4) $$\forall t\;x\in {ℬ}_{x}\subset {\mathbb{R}}^{n}$$

(5) $$\forall t\;u\in {ℬ}_{u}\subset {\mathbb{R}}^{m}$$

(6) $$x(0)=x_{0}$$

(7) $$x(T)\in X_{*}\subset {\mathbb{R}}^{n}$$

where *t*_*s*+1_ = *t*_*s*_ + Δ*t*_*s*_ is the starting time of the phase *s* (with *t*_0_ = 0 and *t*_*S*_ = *T*). In the direct multiple shooting problem a phase *s* corresponds to an interval where the system is simulated using constraint (1b) which makes sure that the motion is dynamically consistent. Phases are connected through the constraints (1d) and (1e) which impose bounds on the state and the control, they are lying, respectively, in admissible set of states $B_{x}$ and in admissible set of controls $B_{u}$. Constraint (1c) enforces balance with respect to the contact model. Breaking and adding contact are usually done at phases junctions because it changes the structure of the dynamics. Constraint (1f) imposes the trajectory to start from a given state (estimated by the sensor of the real robot). Constraint (1g) imposes the terminal state to be in the viable terminal states set $X_{*}$ (Wieber, 2008). The cost (1a) is decoupled ℓ_*s*_(**x**, **u**) = ℓ_*x*_(**x**)+ℓ_*u*_(**u**) and its parameters may vary depending on the phase. ℓ_*x*_ is generally used to regularize and to smooth the state trajectory while ℓ_*u*_ tends to minimize the forces. The resulting control is stable as soon as ℓ_*x*_ comprehends the *L*_2_ norm of the first order derivative of the robot center of mass (CoM), Wieber et al. (2015). Problem (1) is difficult to solve in its generic form. And specifically (1b) is a challenging constraint. Most of the time the shape of the problem varies from one solver to another one only by the formulation of this constraint. The difficulty is due to two main factors: (1) There is a large number of degrees of freedom (DoF). In practice we need to compute 36 DoF for the robot on a preview window with 320 iterations (1.6 s) to take into account the system inertia. (2) The dynamics of the system is nonlinear. Figure 2 depicts the structure of the problem. To be able to solve the whole problem, represented by the full rectangle in Figure 2 researchers often use nonlinear optimization. In this paper we evaluated a resolution of the MPWBC based on the formulation given by Equation (1). In this approach described in Koch et al. (2014), the authors computed a dynamical step-over motion with the HRP-2 robot, but this process can take several hours of computation. So simplifications are necessary, for example Tassa et al. (2014), Koenemann et al. (2015) use simplifications on the contact model. This method is very efficient but not suitable for complex contacts during walking. Seminal works (Kajita et al., 2003b; Orin et al., 2013) show that (1b) can be divided into two parts, the non-convex centroidal dynamics (top horizontal rectangle in Figure 2) (Orin et al., 2013) that includes few DoF, and the convex joint dynamics (vertical rectangle in Figure 2). They optimize for the centroidal momentum on a preview horizon and the next whole body control. They solve first for the centroidal momentum and then for the whole body control. In general the centroidal momentum remains difficult to handle due to its non-convexity. Finally Kajita et al. (2003a), Herdt et al. (2010), and Sherikov et al. (2014) linearize the centroidal momentum which provides a convex formulation of the locomotion problem. In Deits and Tedrake (2014), the problem was formulated has a mixed-integer program (i.e., having both continuous and discrete variables) in case of flat contact. In Mordatch et al. (2012), the same problem was handled using a dedicated solver relying on a continuation heuristic, and used to animate the motion of virtual avatars.

### 2.2. Benchmarking

Different methods exist to benchmark robot control architectures. In del Pobil et al. (2006) the authors argue that robotic challenges offer an efficient way to do so. For example, the results of the DARPA Robotics Challenge published in the Journal of Field Robotics special issues Iagnemma and Overholt (2015) and Spenko et al. (2017), show the different control architecture in a determined context. Each behavior successfully accomplished grants point to the team and the best team wins the challenge. This benchmarking was however costly as the robots had no system to support them in case of fall. In addition, as it is mostly application driven, the challenge provides an overall evaluation of the system integration but not of the independent sub-parts.

For the specific case of motion generation, it has been recently proposed by Brandao et al. (2017) to use a scenario called “Disaster Scenario Dataset.” It allows benchmarking posture generation (solved by the WBC) and trajectory generation (MPWBC) using optimization. A set of problems is proposed by means of foot step locations (*F*_*RF*_, *F*_*LF*_). Using this approach, it is possible to compare algorithms realizing the two functionalities (WBC and MPWBC). The evaluation is realized in simulation using the Atlas robot and the ODE dynamic simulator. This first step is necessary but one step further is required to benchmark a real humanoid platform. For this paper we used a more systematic decomposition of the humanoid bipedal locomotion (Torricelli et al., 2015). Further description can be found in section 3.7. This paper focuses on evaluating the MPWBC and WBC on the Robot Hardware. The Estimator used in this context is important but it is reflected in the stabilization process. The Motion Planning is not evaluated here as the planned motion is always the same or solved at the MPWBC level. The Localization is provided by a motion capture system.

### 2.3. A motivating example: the koroibot project

The work presented in this paper takes its root in the context of the European project Koroibot (http://www.koroibot.eu/). The goal of the Koroibot project was to enhance the ability of humanoid robots to walk in a dynamic and versatile way, and to bring them closer to human capabilities. The Koroibot project partners had to study human motions and use this knowledge to control humanoid robots via optimal control methods. Human motions were recorded with motion capture systems and stored in an open source data base which can be found at https://koroibot-motion-database.humanoids.kit.edu/. With these data several possibilities were exploited:

- Criteria that humans are assumed to minimize using Inverse Optimal Control.
- Transfer from human behaviors to robots given by walking alphabets and learning methods (Mandery et al., 2016).
- Human behaviors safely integrated in robots by means of optimal controllers.
- Design principles derived for new humanoid robots (Clever et al., 2017; Mukovskiy et al., 2017).

**Figure 3:**
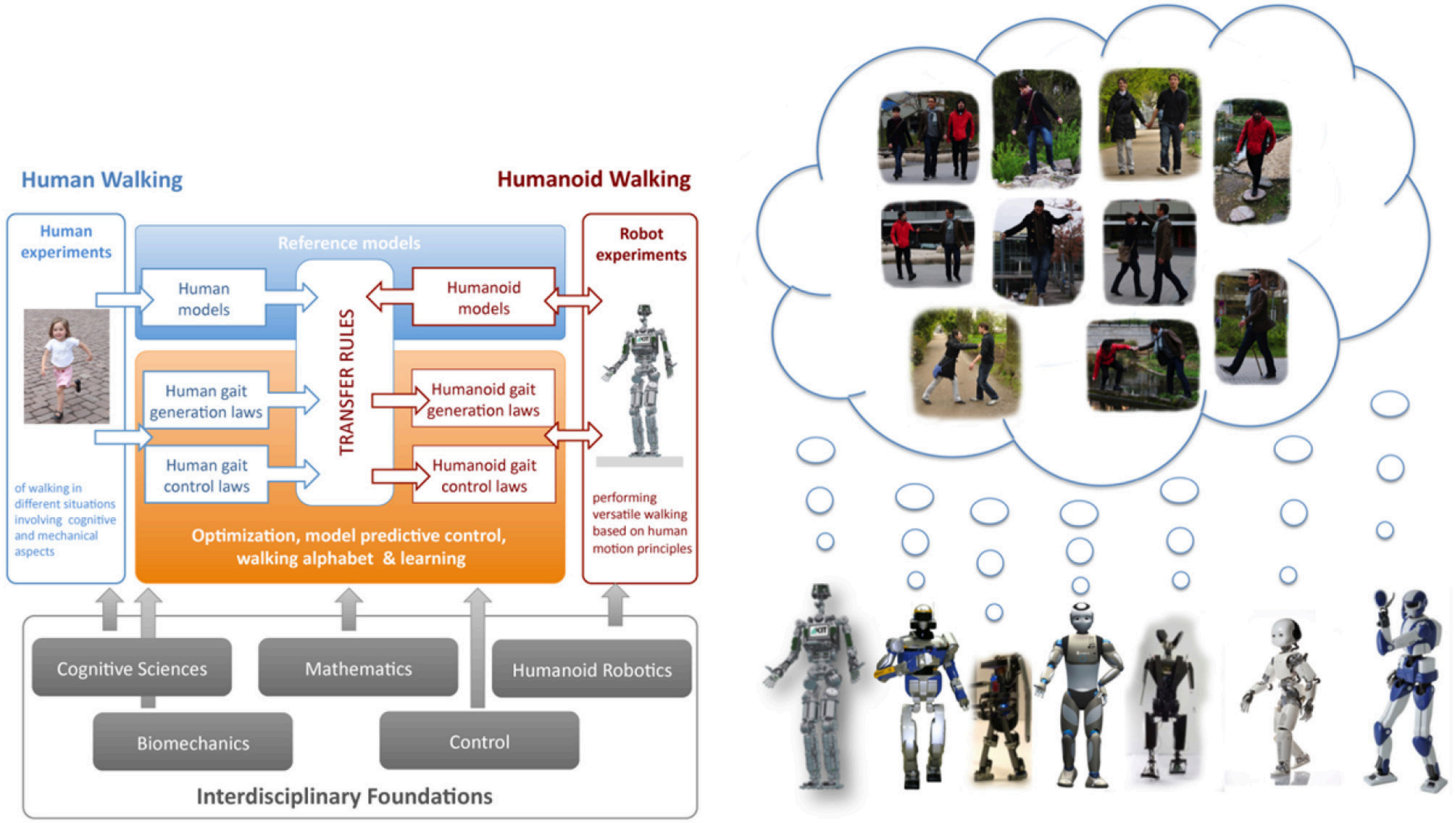
**(Left)** Graphical representation of the scientific approach of the Koroibot project. **(Right)** View of the humanoid robot used in the Koroibot project dreaming of human walking capabilities (Pictures taken from http://www.koroibot.eu).

**Figure 4:**
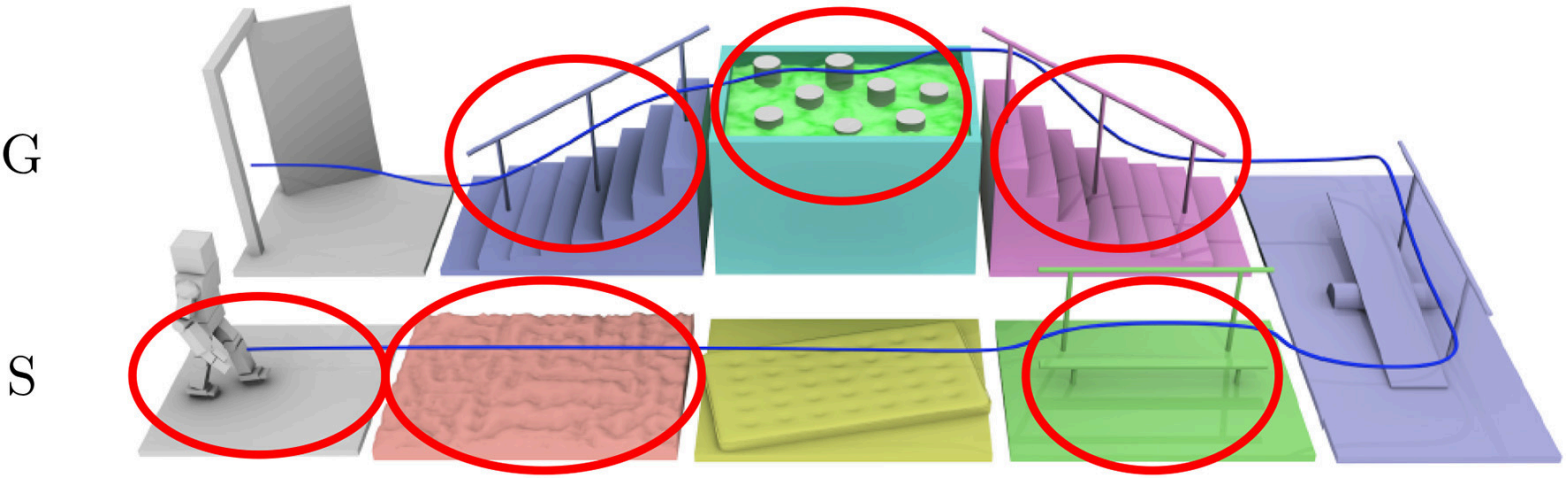
Challenges of the Koroibot project. In red the challenges chosen by the LAAS-CNRS.

In order to evaluate the progress of the algorithms at the beginning and at the end of the project, a set of challenges focusing specifically on walking were designed (see Figure 4). Figure 3 (right) shows all the robots hosted by the Koroibot partners. Each team owning a robot had to perform some of these challenges considering the current and the potential state of their robots and controllers.

### 2.4. The key performance indicators (KPI)

**Figure 5:**
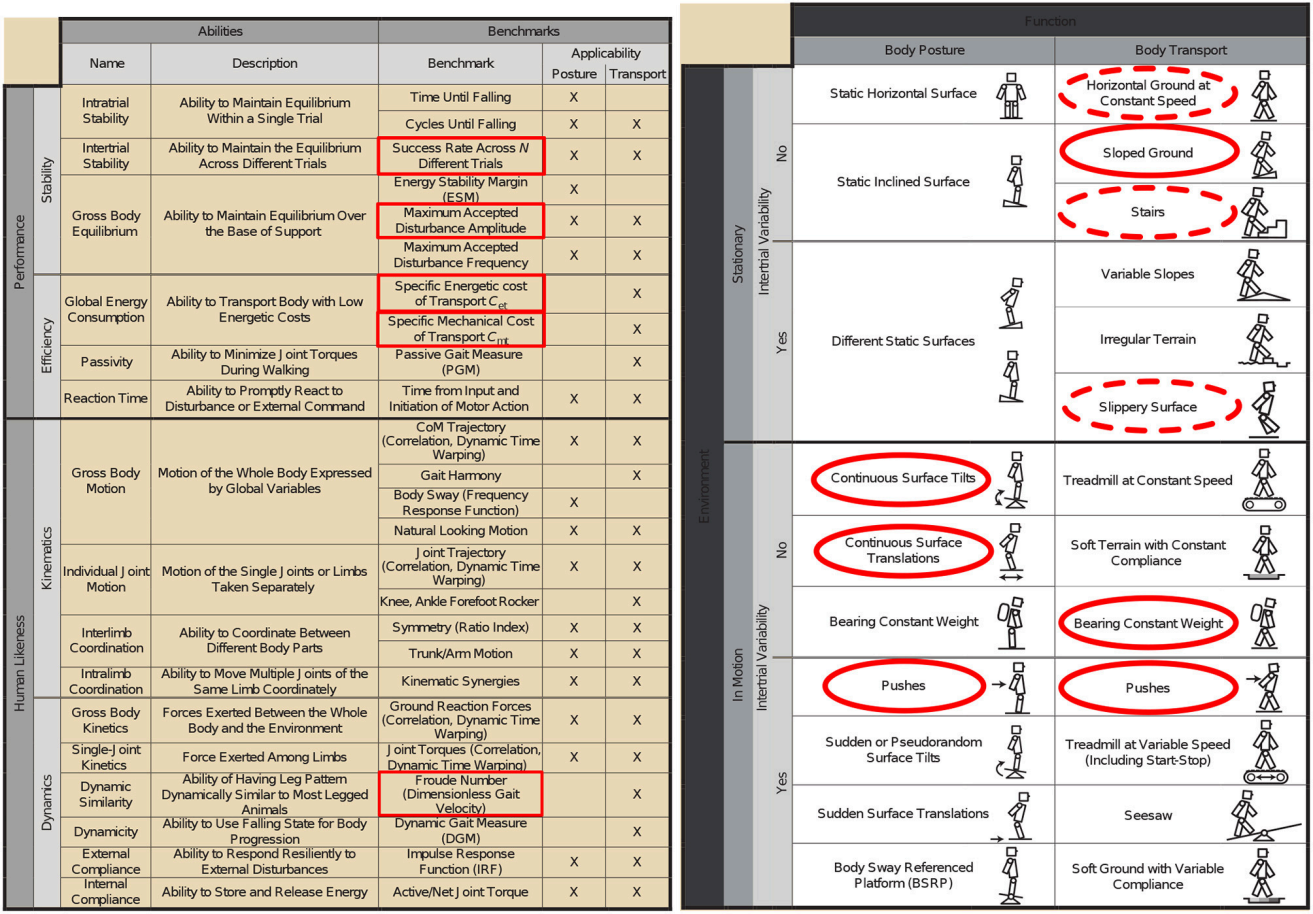
**(Left)** Performances indicators, **(Right)** motor skills considered in the benchmarking scheme. This scheme is limited to bipedal locomotion skills. The concept of intertrial variability represents modifications of the environment between trials. (dashed) motor skills evaluated in Naveau (2016) (not dashed) motor skills evaluated in this paper.

In this context and in collaboration with the H2R project, a detailed set of key performance indicators (KPI) have been proposed (Torricelli et al., 2015). These KPI try to capture all the bipedal locomotion patterns. Specific sub-functions of the global motor behaviors were analyzed (see Figure 5, right). The results are expressed as two different sub-function sets. First, the sub-functions associated with the body posture task without locomotion. Second, the same sub-functions but including the robot body transport. The initial condition may vary depending on the experiment to perform. This is the idea of the intertrial variability. The sub-functions are also classified by taking into account the changes in the environment or not. Each of these functions can be evaluated for different robots using the criteria depicted in Figure 5 (left). The performances are classified into two sub categories, quantitative performances and human likeness. In addition, information in the last two columns indicate whether the criteria is applicable on a standing task or on a locomotion task. Again, all the teams owning a robot had to perform an evaluation of these KPI, considering the current and potential state of their robots and controllers.

### 2.5. The work done in the koroibot context

In the Koroibot context the Gepetto team evaluated the KPI one the robot HRP-2 (second robot from the left in (Figure 3, right). Among the challenges presented in Figure 4, we considered the following ones:

- walking on a flat ground,
- walking on an uneven ground,
- walking on a mattress,
- walking on a beam without handrail,
- climbing a stair case with/without handrail,
- walking on stepping stones,
- going down a stair case without handrail,

They are depicted by red circles in Figure 4. In addition to these challenges we added the perturbation rejection. Considering the selected challenges we picked the following KPI:

- horizontal ground at constant speed,
- stairs,
- bearing constant weight (the robot's own weight)

while considering the following motor-skills:

- success rate across N different trials,
- mechanical energy,
- mechanical plus electrical energy,

All these choices are shown in Figure 5 by red ellipses in the table. The mathematical details and results are presented below in section 3.7.

## 3. Materials and methods

**Figure 6:**
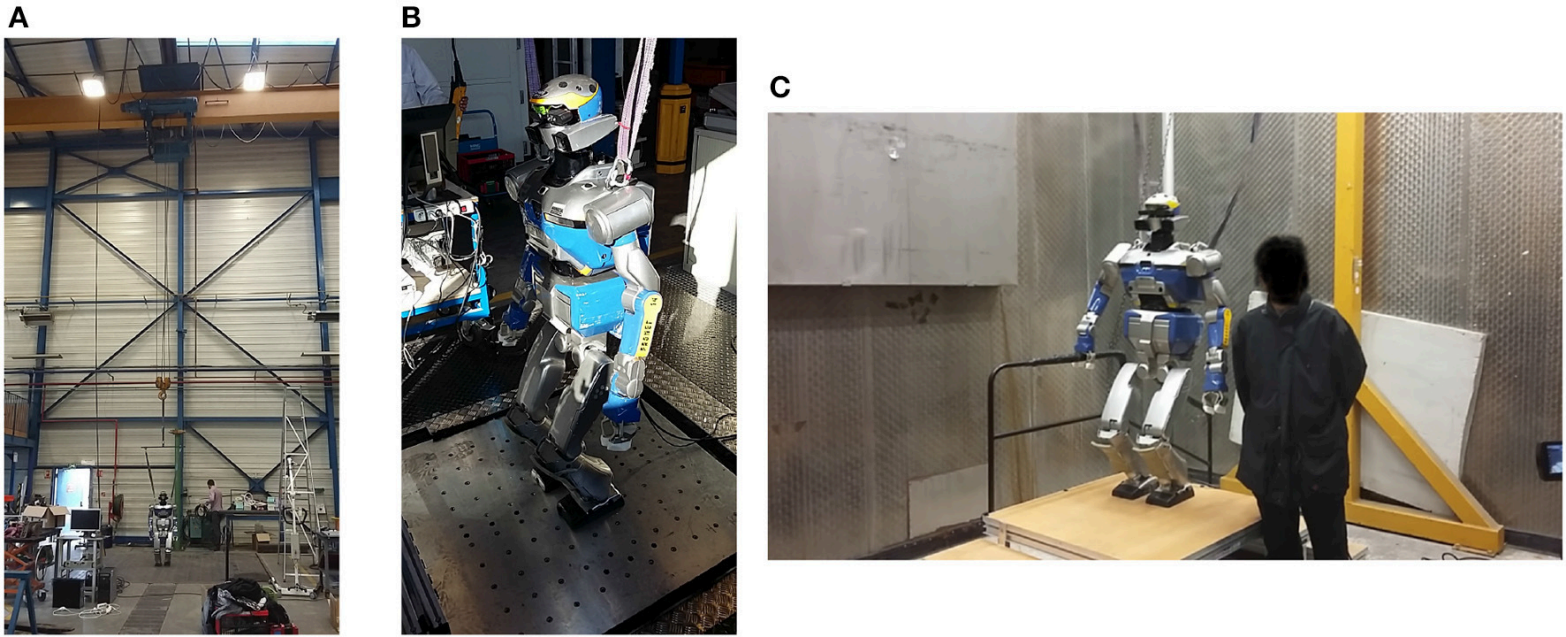
Pictures of the experimental setup at LNE **(A)** the robot hang up to walk on a slope **(B)** the translational plate **(C)** the temperature-controlled chamber (end of the robot climbing 15 cm at 10°C).

The experimental setups used to compute each of the performance indicators given in section 3.7 are described in this section. The motor skills given in Figure 5 and their implementation are also presented. In addition, the algorithms used to perform the different tests are depicted in section 3.8 and illustrated in Figure 6.

### 3.1. Different temperatures

The LNE is equipped with temperature-varying rooms which allowed us to measure some of the performance indicators at various temperatures ranging from 5°C to 45°C. In this way, we evaluated the robustness and limits of our robot with respect to the performance indicators in different environmental conditions. It appeared that the robot behavior deteriorates at low temperatures. At 5°C it is not possible to perform the calibration procedure as the robot could not move. At 10°C the friction is sufficiently low such that the robot could move. Another phenomenon occurs above 40°C after few motions due to internal temperature build up: thermal protection prevents the robot from moving if the temperature is too high. In this room, apart from these extreme cases, the motions and indicators measurements have been performed as expected on a flat ground or on the staircase testbed of the Koroibot project. This staircase is made of 4 15 cm high stairs and a top platform. The dimension of one stair case is 1m × 0.25m × 0.05m.

### 3.2. Tilted surfaces

In the context of the body skills in motion, we considered tilting surfaces. This was tested with the stabilizer commercially available with HRP-2. The setup is a platform which can be tilted upward and downward on one side with a hydraulic actuator. The surface was tilted continuously until the robot fell off. On the other hand, we tested walking algorithms with different angles (pointing up or down) until the robot fell down. Tests were realized with the robot pointing down, pointing up and across the slope. In Figure 5 this test corresponds to Body Posture—Continuous Surface Tilts.

### 3.3. Horizontal translations

We used a mobile plate controlled in the horizontal plane to perform continuous oscillating surface translations at various frequencies and various amplitudes. The platform was moved by a hydraulic actuator. The aim was to find the frequency and the amplitude that the controlled robot is able to sustain. In Figure 5 this test corresponds to Body Posture—Continuous Surface translations.

### 3.4. Bearing

In order to test the robot capability to bear weights, we loaded it with additional masses (bags of 5–15 kgs) in such way that its balance is maintained. This approach is a bit limited as they are several ways to bear a weight. Indeed it can be done with a backpack, in collaboration with someone, or by holding the object against its chest. Each of this approach comes with its own specific constraint. In order to avoid such constraints, we decided to take the simplest choice and hang soft weights on the front and the back of the robot chest. In Figure 5 this test corresponds to Body Transport—Bearing Constant Weight.

### 3.5. Pushes

This paragraph presents the pushes experiments. We tried to find the sufficient force to make the robot fall down. This was achieved by using a stick on top of which was fixed a force sensor displaying the maximum force measured during an experiment. The sensor used was a HBM 1000 N of type u3 together with a HBM Scout 55 amplifier. The experience was realized while the robot was standing and walking. The force was applied in the sagittal and frontal planes until making HRP-2 fall. The force was applied from behind the waist of the robot. This part of HRP-2 was made specifically soft to support impacts. The walking part is the most difficult in terms of repeatability as the robot might be in different foot support and therefore more or less stable depending on the configuration. In Figure 5 this test corresponds to Body Posture—Pushes and Body Transport—Pushes.

### 3.6. Data

A CAD model of the staircase used is available on the github repository where all the log of the experiments are also present: https://github.com/laas/koroibot_KPI. All the computations performed on the logs and implementing the key performance indicators are available here: https://github.com/laas/EnergyComputation.

### 3.7. Key performance indicators (KPI)

In this section the performance indicators used to evaluate the humanoid robot HRP-2 are described. They are mostly based on the work proposed in Torricelli et al. (2015). In the Koroibot project we used key performance indicators (KPI) to analyze the behavior of the robot at the beginning and at the end of the project. These results lead us toward the improvements to be made. In 2013 the algorithm mostly used and implemented on HRP-2 in LAAS-CNRS where the walking pattern generators described in Morisawa et al. (2007) and Herdt et al. (2010). The performance indicators chosen were:

- The execution time *T*_*M*_ = *t*_*end*_−*t*_*begin*_, where *t*_*begin*_ is the time at which the sum of the norm of the motor axis velocities reaches 6*rads*^−1^ for the first time in the log and *t*_*end*_ is when the sum of the norm of the motor axis velocities passes below 0.5*rads*^−1^.
- The walked distance, being the distance between the final base position and the initial one. The base pose is reconstructed using odometry with the joint positions only. The drift of this odometry is 8 cm over 3.6 m during a straight walk.
- The success rate, being the number of time a specific task could be performed without falling, over the total number of trials of the task.
- The maximum tracking error from the planned trajectory,
  (8) $$\begin{array}{l} TrackingError\left(t\right)=\overset{t+0.1}{\underset{t}{\int }}|q^{ref}-\tilde{q}|dt/0.1\\ MaxTrackingError=\underset{t}{\max}\left(TrackingError\left(t\right)\right) \end{array}$$
  with *TrackingError* being the average normed difference between the desired joint trajectory (*q*^*ref*^) and the joint pose measured from the encoder ($\tilde{q}$) during 0.1 s starting at time *t*. And *MaxTrackingError* being the maximum value of the *TrackingError* function.
- The mechanical energy consumed normalized over the walking distance *D* and the execution time *T*_*M*_.
  (9) $$\begin{array}{l} E_{mechanical}=\overset{t_{end}}{\underset{t_{begin}}{\int }}|\tau \omega |dt/\left(T_{M}D\right) \end{array}$$
  with *E*_*mechanical*_ being the integral over time of the mechanical power, τ being the torques applied at the robot joints and ω being the velocity of the robot joints.
- The electrical energy dissipated by the motor resistance normalized over the walking distance *D* and the execution time *T*_*M*_,
  (10) $$\begin{array}{l} E_{motor\;resistance}=\int_{t_{begin}}^{t_{end}}R\;i^{2}dt/(T_{M}\;D)\\ \;=\int_{t_{begin}}^{t_{end}}R\;k_{c}^{2}\;\tau^{2}dt/(T_{M}\;D) \end{array}$$
  with *E*_*motorresistance*_ being the integral over time of the electric power dissipated, *R* being the motor resistances, *k*_*c*_ being the electric motor torque constant and τ being again the torques applied at the robot joints.
- The total energy consumed during the walking distance *D* and the execution time *T*_*M*_,
  (11) $$\begin{array}{l} E_{total}=E_{mechanical}+E_{motorresistance}+E_{electronics} \end{array}$$
  with *E*_*total*_ being the sum of the energy consumed by the system normalized over the walking distance *D* and the execution time *T*_*M*_, and *E*_*electronics*_ being the energy consumed by the on-board electronic cards. *E*_*electronics*_ is neglected in this study so:
  (12) $$\begin{array}{l} E_{total}=E_{mechanical}+E_{motorresistance} \end{array}$$
- The mechanical cost of transport and the total cost of transport,
  (13) $$\begin{array}{ll} E_{mechanicalcosttransport} & =\overset{t_{end}}{\underset{t_{begin}}{\int }}|\tau \omega |dt/\left(mgD\right)\\ E_{totalcosttransport} & =\left(\overset{t_{end}}{\underset{t_{begin}}{\int }}|\tau \omega |dt+\overset{t_{end}}{\underset{t_{begin}}{\int }}Rk_{c}^{2}\tau^{2}dt\right)/\left(mgD\right) \end{array}$$
  with *E*_*mechanicalcosttransport*_ and *E*_*totalcosttransport*_ being, respectively, the mechanical and total cost of transport, *m* being the total mass of the robot, and *g* = 9.81ms^−2^ the gravity constant.
- The Froude number,
  (14) $$\begin{array}{ll} F_{r} & =\frac{v}{\sqrt{gl}}\\ v & =\frac{D}{T_{M}} \end{array}$$
  where *v* is the robot center of mass mean velocity along the horizontal plane and *l* is the leg length. This number represents the ratio between the kinetic energy and the potential energy. It can also be interpreted as an indicator on the stepping frequency.

**Figure 7:**
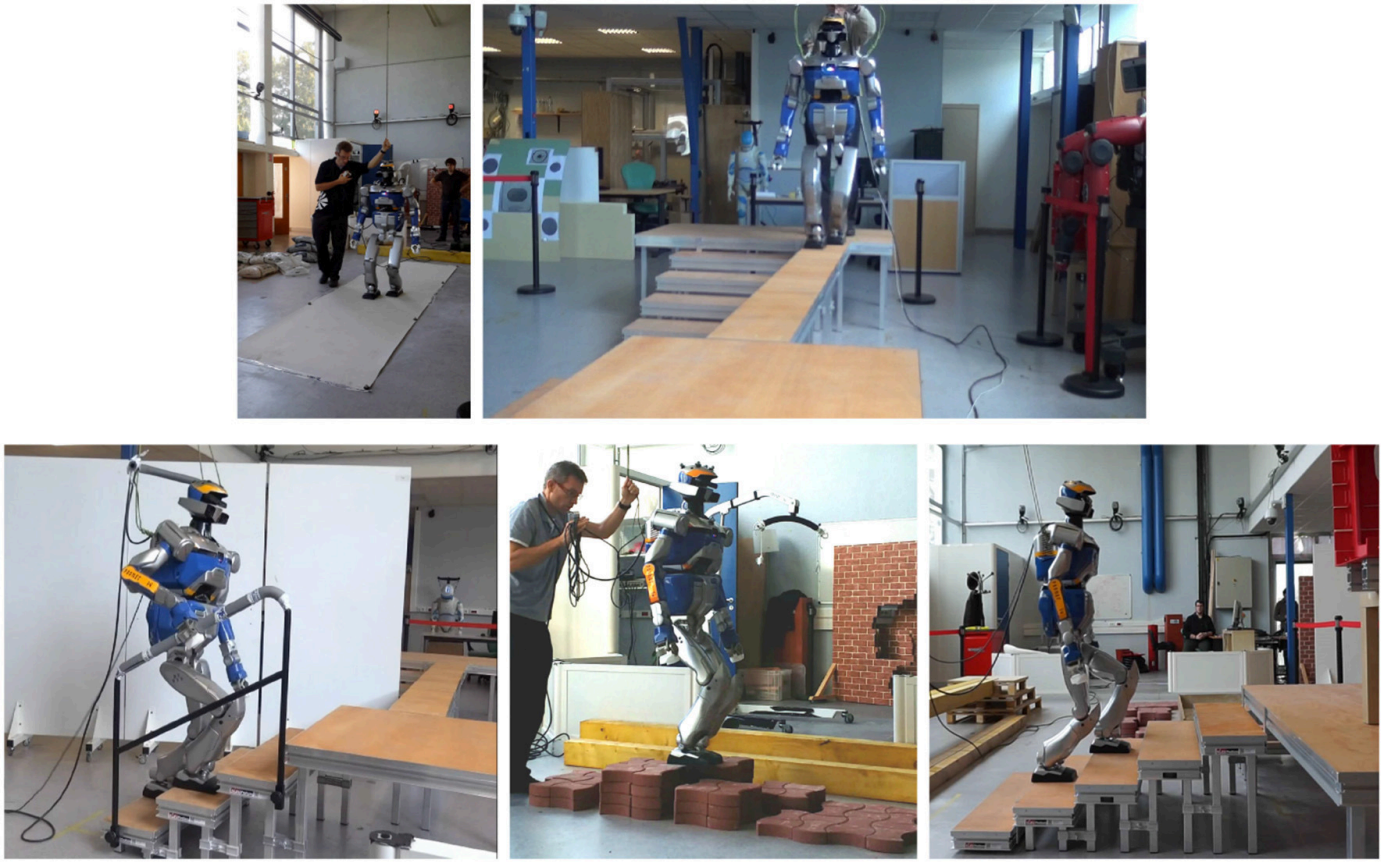
Sample of the experimental setup of the Koroibot project in LAAS-CNRS.

The trajectories were generated off line and repeatedly played on the robot to analyze their robustness. Views of the experimental setups are given in Figure 7.

### 3.8. Motion generation for humanoid robot locomotion

This section explains the links between the motion generation architecture depicted in Figure 1 and the Key Performance Indicators given in the section 3.7. The set of functions entitled body posture, depicted in Figure 1 (right), represents the behavior which is provided by what is called a whole-body controller. It consists of two parts:

- an estimator, which provides the orientation of the robot with respect to the gravity field and the positions of the end-effectors in contact with the environment.
- a whole-body controller which guarantees that the robot balance is maintained with respect to *c*^*ref*^, *f*^*ref*^ and possibly a *q*^*ref*^.

In this paper we have evaluated independently only one whole body motion controller. It is the stabilizer provided by Kawada Inc. We give detailed performances evaluation of this controller in the experimental part of this paper. It was described in various paper such as Kajita et al. (2007) and Kajita et al. (2001).

The set of function entitled body transport, depicted in Figure 1 (right) in this paper, are four CDPG and one MPWBC. The four CDPG evaluated in this paper are the following ones: Carpentier et al. (2016), a multi-contact centroidal dynamic pattern generator used to climb stairs with given contact positions, Kajita et al. (2003a), the original walking pattern generator implemented by Shuuji Kajita with given foot steps, Morisawa et al. (2007), an analytical walking pattern generator allowing immediate foot step modifications, Naveau et al. (2017), a real time nonlinear pattern generator able to decide autonomously foot-steps positions. In each case the goal of the CDPG is to generate a center of mass trajectory and the foot-steps trajectories. For Kajita et al. (2003a), Naveau et al. (2017), and Morisawa et al. (2007) a dynamical filter is used to correct the center of mass trajectory to improve the dynamical consistency of the motion. In each case, a whole body motion generator (not to be confused with a whole body motion controller) is used without feedback to generate the reference position *q*^*ref*^, and the desired *z*^*ref*^ which are then sent to the stabilizer. For Naveau et al. (2017) and Morisawa et al. (2007) we used the stack of tasks described in Mansard et al. (2009) as a Generalized Inverse Kinematics scheme. In Carpentier et al. (2016) a Generalized Inverse Dynamics was used to generate the reference value for *q*^*ref*^ and *c*^*ref*^. The MPWBC provides the controls directly. The one used is from Koch et al. (2014) using the Muscod-II Diehl et al. (2001) nonlinear solver.

## 4. Results

In this paragraph we present the numerical results obtained from the computation of the KPI explained in detail in section 3.7 for each set of experiments. As a reminder the list of the KPI is recalled:

- walked distance,
- success rate,
- max tracking error,
- duration of the experiment,
- mechanical joint energy,
- actuators energy,
- cost of transport,
- mechanical cost of transport,
- Froude number.

A video displaying a mosaic of all the experiments is available at the following URL: https://www.youtube.com/watch?v=djWGsb44JmY&feature=youtu.be or as a Supplementary Material on the editor site of this paper.

### 4.1. Climbing stairs

#### 4.1.1. Stairs of 10cm

**Figure 8:**
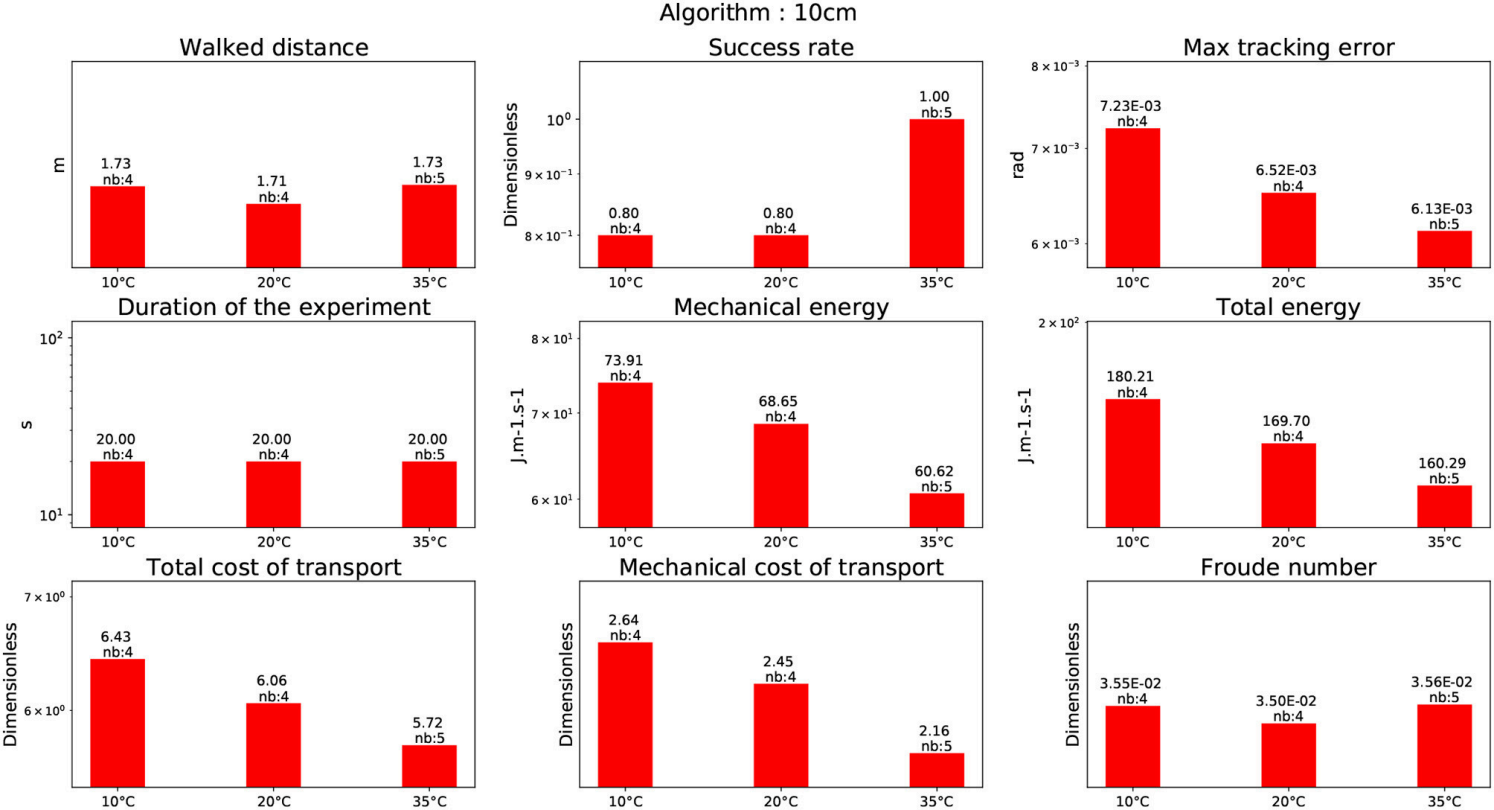
Climbing 10cm stairs without handrail.

**Figure 11:**
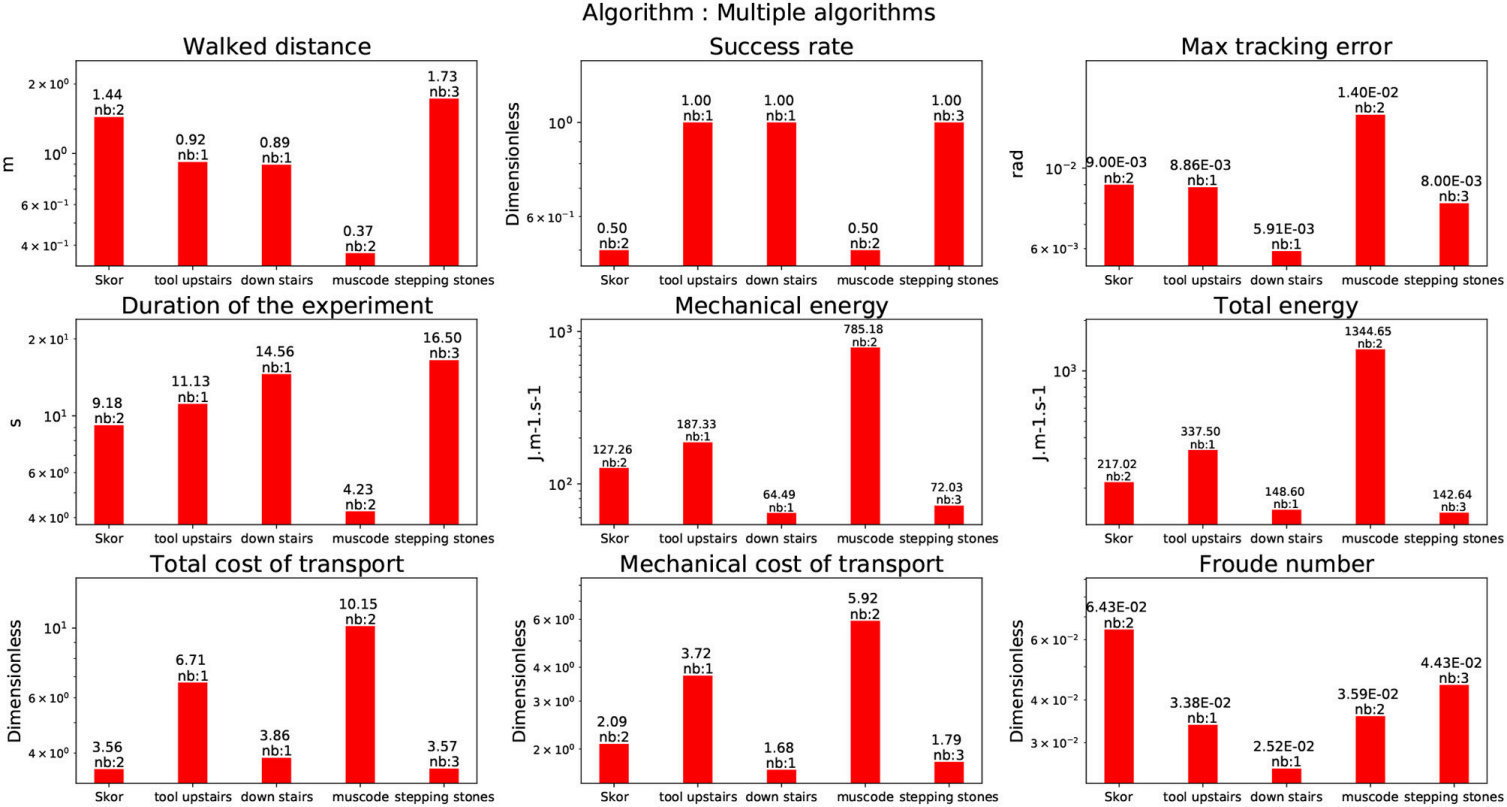
Multiple algorithms: Slopes at 5 degrees using Kajita algorithm (Skor), going up with a tool on a wooden pallet 10 cm (tool upstairs), going down on a wooden pallet 10 cm (down stairs), going over an obstacle solving an OCP approach (Muscod), stepping on a interlocking paving stones (stepping stones).

In this experiment, the humanoid robot HRP-2 is climbing stairs of 10cm height without any handrail. The difficulty of this task is that the robot has to perform quite large steps and vertical motion. For this reason, the robot is climbing one stair at a time, which means that the robot puts successively one foot on the next stair and the other one on the same stair. This avoids a too large joint velocity that the robot could not track. Morisawa et al. (2007) CDPG was evaluated at the beginning of the project although the variation of height violates the assumption of the cart-table model. But thanks to the dynamical filter the motion generated was dynamically consistent so that the stabilizer could cope with the situation. Because this experiment was not performed at the LNE (it was done 3 years before) it was not possible to control carefully the room temperature but the test was performed at 20°C. The KPI results can be seen in Figure 11 (tool upstairs). The other test was performed at the end of the project using the CDPG (Carpentier et al., 2016). This time the CDPG took into account the center of mass height variation but not the whole body motion. The stabilizer should theoretically have less trouble to compensate for the simplifications made. For Carpentier et al. (2016) three different temperatures were tested: 10°C, 20°C, and 35°C. The numerical results are depicted in Figure 8. Interestingly, the temperature level has a direct impact in terms of mechanical cost as it diminishes with the increase in temperature. It is reflected in the tracking error. This intertrial variation does not come from the change of reference trajectory as it is strictly the same for every trial. There is a level of adaptation due to the stabilizer, but each temperature has been tested at least 4 times. A possible explanation is the fact that the grease in the harmonic drives generates less friction at higher temperature. As the cost of transport is dimensionless it allows the two motions to be compared regardless of their duration. It is then interesting to see that the cost of transport in Figure 11 (tool upstairs) and in Figure 8 (10°C) are very similar. And that, at the same temperature, the total cost of transport for Carpentier et al. (2016) CDPG is 9.6% better (from 6.71 to 6.06). One explanation is that the motion from Carpentier et al. (2016) CDPG being more dynamically consistent, the stabilizer consumes less energy to compensate for the model simplifications.

#### 4.1.2. Stairs of 15cm

**Figure 9:**
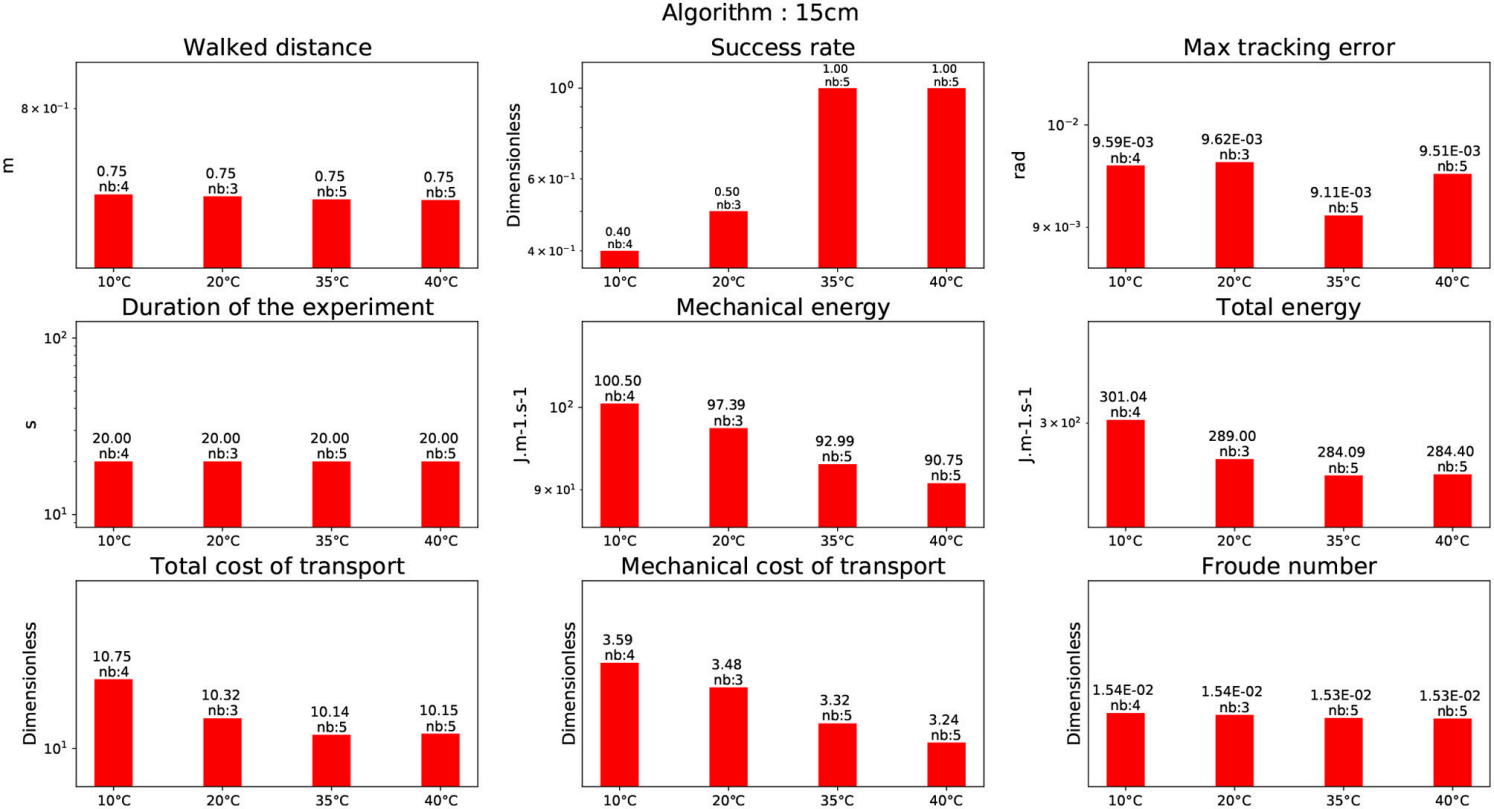
Climbing 15 cm stairs with a handrail.

In this experiment, the humanoid robot HRP-2 is climbing stairs of 15cm height using a handrail. In addition the robot is not using any stabilization algorithm, because there are non-coplanar contacts. In this setup the Morisawa et al. (2007) CDPG has to be used without handrail because of the model simplifications. Trials have therefore been done using a WBC (described in Mansard et al., 2009) without the handrail. The results show that the current demanded by the motors went up to 45*A*. And because the HRP-2 batteries cannot provide more than 32*A*, all trials failed. This is the reason why the results are not shown in this study. Nevertheless, tests using the handrail could be performed with Carpentier et al. (2016) CDPG. The corresponding results are depicted in Figure 9. It confirms that the energy is decreasing with the increase of temperature without the stabilizer. Note that the energy spent by the robot is clearly higher than for the experience on the 10 cm stairs, i.e., a 36% of increase of the energy for walking.

#### 4.1.3. Stepping stones

In this experience, the humanoid robot HRP-2 had to walk up and down on stairs made of red interlocking paving stones. Between each stair there is a height difference of ±5cm. The CDPG described in Morisawa et al. (2007) was used. this test is slightly different from the previous experiments because the robot cannot put his two feet on a same level surface (contrary to a stair step). To cope with this, the generated trajectories had to always change the height of the next support foot. As the paving stones were always slightly moving due to the robot weight, the balance was difficult to obtain in a reliable way. As indicated in the graph depicted in Figure 11, despite a success rate of 1, the tracking error reaches a level (8*e*^−03^*rad*). This tracking error is greater than the one obtained during the 10 cm climbing experiment at 10°C but lower than the one obtained during the 15 cm climbing experiment at 35°C (which is the lowest for this temperature and the CDPG). A possible explanation of why the energy consumption is greater than during the 10 cm climbing stairs might be the instability of the stones and the fact that in this experiment the robot climb the stairs in a human fashion, i.e., not one stair at a time.

### 4.2. Walking on a beam

**Figure 10:**
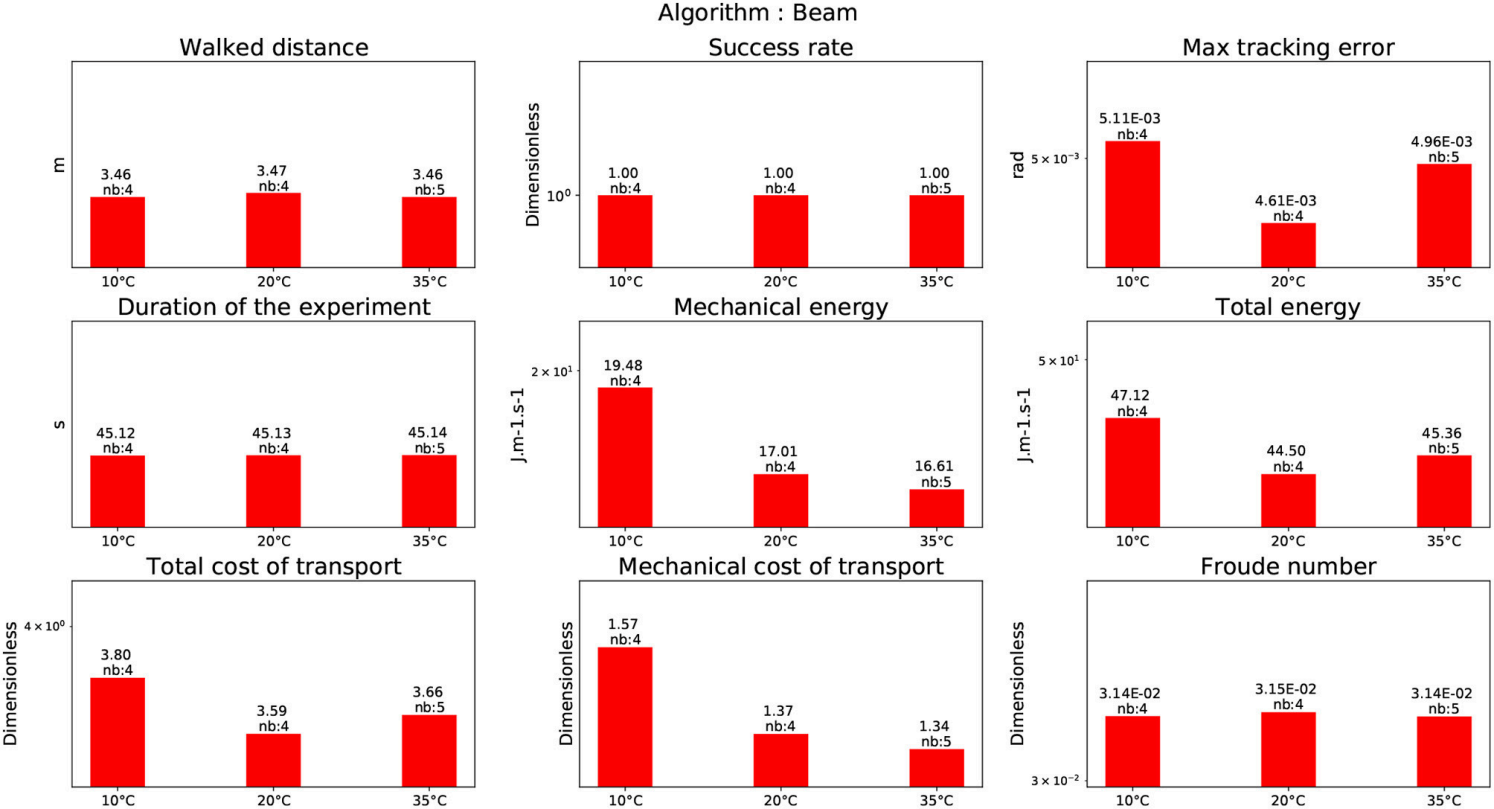
Walking on a beam.

This experiment was realized using the CDPG Morisawa et al. (2007). In this experiment the humanoid robot HRP-2 is walking on a beam. Initially, the experiment success rate on a real beam was around 20%. This rate was improved to achieve a 90% success rate, thanks a new implementation of the dynamical filter presented in Kajita et al. (2003a). It reduced the drift which is important as the beam length is 3*m* long. This could probably be improved by a proper vision feed-back. However, in these experiments, the robot walked on a normal ground as if it was on a beam. The reason is the absence of a beam in the temperature-controlled room. Even though the foot step location is discarded, the balance problem is exactly the same. Here, the success rate is 1. The corresponding result is depicted in Figure 10.

**Figure 12:**
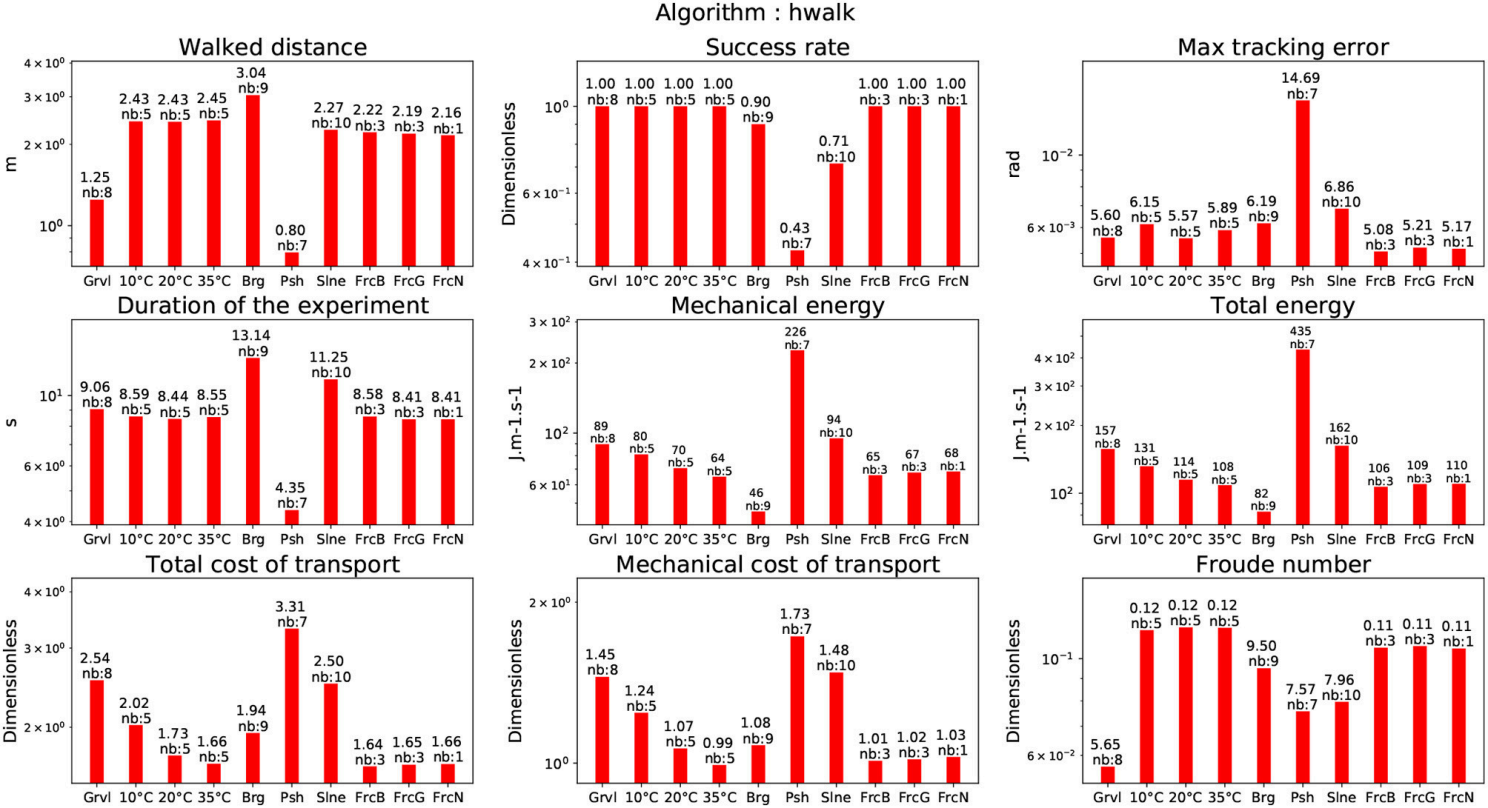
Straight walk with Kajita's walking pattern generator (Kajita et al., 2003a).

To perform the motion on a limited bandwidth (beam), the robot has to execute faster motions with its legs in order to place its foot ahead the previous one. It is emphasized by the increase of the cost of transport compared to normal straight walking (see Figure 12). Though the robot's legs are moving faster, the step frequency is lowered compared to a normal walking in order to keep the joint velocities in the feasible domain. This is reflected by the fact that the Froude number is around 35% less than during a straight walking (see Figure 12).

### 4.3. Straight walking on flat ground

#### 4.3.1. Temperatures

**Figure 13:**
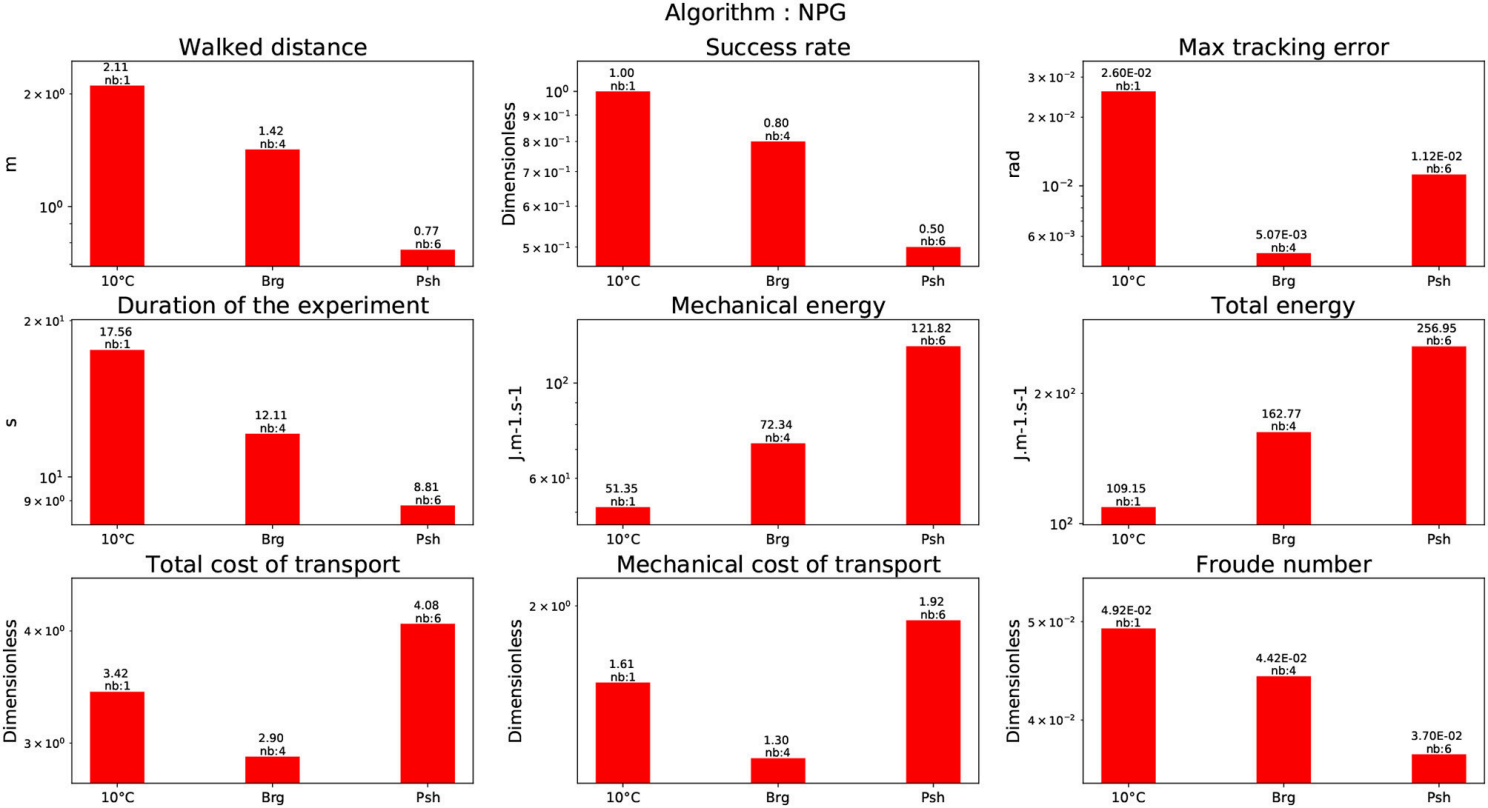
Straight walk with the walking pattern generator described in Naveau et al. (2017).

In the temperature-controlled room the humanoid robot HRP-2 is performing a 2m straight walking following the implementation of Kajita et al. (2003a). The corresponding result is depicted in Figure 12. Note that the energy with respect to the temperature is following the same trend as for the experiments on the stairs and on the beam. We also tested the algorithm (Naveau et al., 2017) at 10°C. The total cost of transport is higher than the algorithm (Kajita et al., 2003a) at the same temperature but lower than the one used for walking over the beam. It is however strongly less than the total cost of transport for climbing stairs at 10°C. The fact that the energy cost is higher for Naveau et al. (2017) than for Kajita et al. (2003a) at the same temperature is that Naveau et al. (2017) (illustrated in Figure 13) provides a higher range of motion but the generated motions are closer to the limit of the system, so the stabilizer spends more energy to compensate for this.

#### 4.3.2. Bearing weights

We made the humanoid robot HRP-2 walk while bearing weights at ambient temperature between 15° and 19°. The two algorithms Kajita et al. (2003a) and Naveau et al. (2017) were tested. The robot was able to walk while carrying up to 14 kg with the two algorithms. Note that, as expected, the effort to compensate for the additional weight reflects in the cost of transport.

#### 4.3.3. Pushes

We performed pushes in the lateral direction and in the frontal direction while the robot was walking along a straight line. The two algorithms Kajita et al. (2003a) and Naveau et al. (2017) were again tested. In our case, the tested algorithm was not able to modify its foot-steps according to the pushes contrary to the impressive work by Takumi et al. (2017). For this specific set of experiments with push from the back, the robot was able to sustain forces from 31*N* to 47*N*. Pushes applied in the lateral plane were varying between 23*N* and 40*N*. For Kajita et al. (2003a), the cost of transport has a value of 3.31 similar to the one obtained when walking on the beam. It is lower than the cost of transport for climbing stairs. The cost of transport for Naveau et al. (2017) is of 4.08. For both algorithms pushes are among the most consuming behaviors. It is due to the stabilizer action to compensate for the perturbation.

#### 4.3.4. Slopes

The robot walked on a straight line while being on a slope of various inclinations ([1°−3.0°]) -and with two possible directions (upward or downward). The two algorithms Kajita et al. (2003a) and Morisawa et al. (2007) were tested. For Kajita et al. (2003a) the cost of transport is higher than for standard straight walking but far less than during the pushes. For Morisawa et al. (2007) the cost of transport is higher than when performing the pushes with Kajita et al. (2003a) approach and is at the same level than the beam test. It can be explained by the fact that when the experiment has been realized the dynamical filter was not used. Therefore the stabilizer had to compensate for the discrepancy between the motion dynamics and the reference given by the center of pressure. An algorithm able to estimate the ground slope and adapt the walking pattern to it would probably increase the efficiency of this motion.

#### 4.3.5. Frictions

The robot walked on carpets with different textures including different friction coefficients. In this case, we did not see any consequences with the CDPG (Kajita et al., 2003a). This is probably due to the particular coating of HRP2 soles used, they might have avoided foot slippage. which is one way to affect the friction coefficient. A possible extension of this work would be to use more slippery ground. But a proper way to handle such case is to implement a slip observer such as it was done (Kaneko et al., 2005).

#### 4.3.6. Uneven terrain

The robot walked over gravels of calibrated size. We tested several diameters with the CDPG (Kajita et al., 2003a). The robot was able to walk on gravels of size up to 8 mm. Beyond this size, the robot was falling. Note that in Figure 12 the cost of transport is slightly more expensive than for classical straight walking at nominal temperature, but not much than walking at 10°C. It is far less expensive than climbing a slope or counteracting pushes. As expected it has no impact on the frequency of the footstep as can be reflected by the Froude Number.

#### 4.3.7. Walking over an obstacle

We have computed the same performance indicators to achieve the task described in Koch et al. (2014) in the frame of the Koroibot project. This strategy is quite different from the others as it implements a MPWBC under the formulation of an Optimal Control Problem given by Equation (1). The solution of this problem was computed by the Muscod-II (Diehl et al., 2001) solver. As the solver is trying to maximize a solution which is not on a reduced space (the centroidal dynamics for the previous algorithms), but on the whole robot, the solution found is close to the limits of the robot in terms of joint position, velocity, acceleration and torques. This is reflected in the cost of transport which is very high, 10.15, almost as high as for climbing the stairs of 15 cm (see Figure 11, Muscod).

### 4.4. Stabilizer

**Figure 14:**
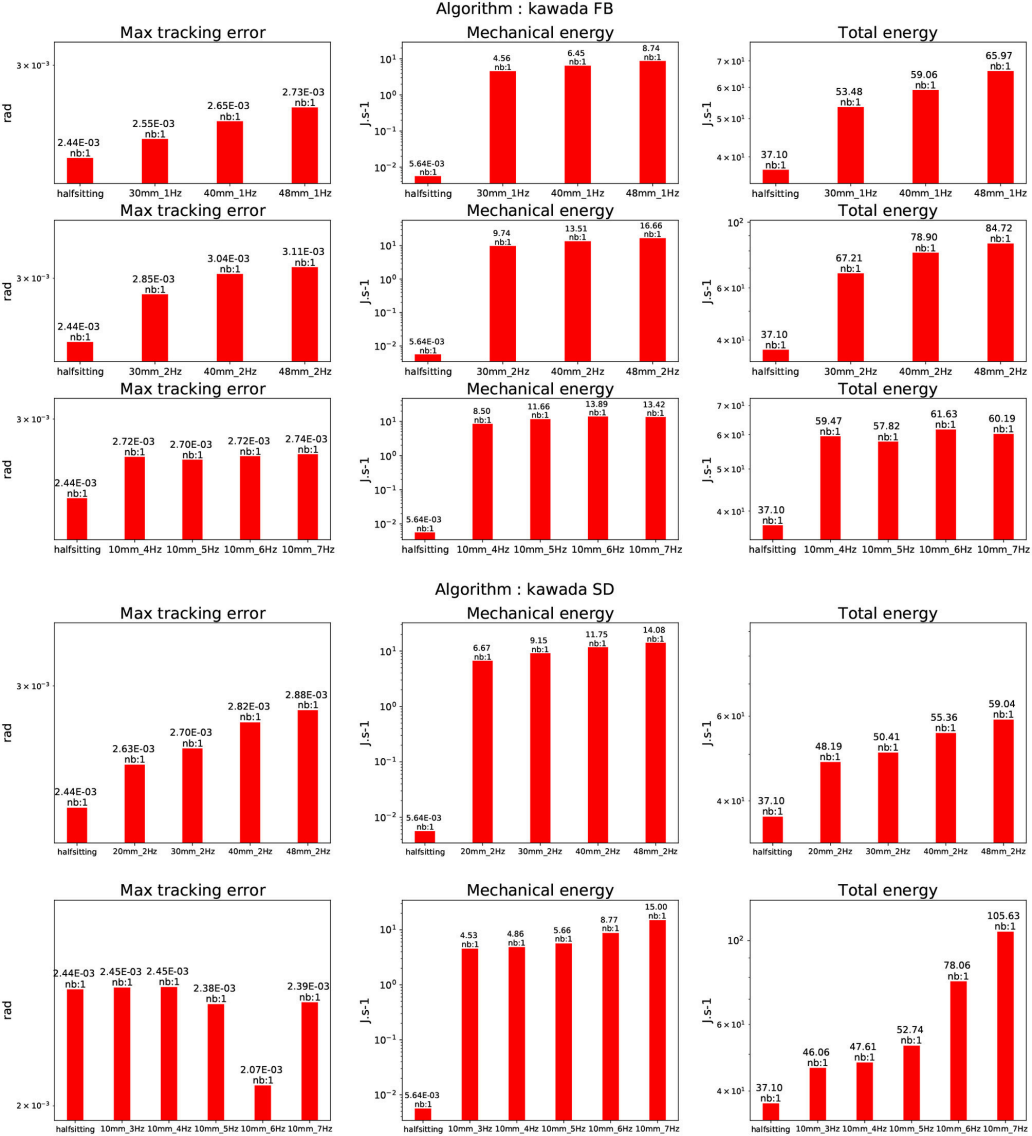
Evaluation of the stabilization algorithm described in Kajita et al. (2007) and Kajita et al. (2001). The upper figure shows the results along the sagittal plane, whereas the lower figure depicts the results along the perpendicular plane.

The stabilizer described in Kajita et al. (2007) and Kajita et al. (2001) was extremely resilient during all the tests. A horizontal testbed platform was used to generate oscillations along the sagittal plane and the perpendicular plane at 1 and 2 Hz at various amplitude [10, 20, 30, 40, 48] in mm. Along the sagittal plane at 40 and 48 mm for both frequencies the feet of the robot were raising up. In the perpendicular plane at 40 and 48 mm for both frequencies the overall robot rotated of about 15° and 20°. It was also tried to increase the frequency for a given amplitude of 10 mm. In the sagittal plane, the robot was able to reach 7 Hz without falling. In the perpendicular plane at 7 Hz the robot was making violent oscillations (without falling) reaching mechanical resonance. The trial was subsequently stopped. The results are depicted in Figure 14. We can clearly see that for the oscillation in the perpendicular plane the increase of total energy is following an exponential curve, compared to the same experience in the sagittal plane. This clearly shows that the resonance frequency of the system was reached as it can be seen in the video available at the following location https://www.youtube.com/watch?v=djWGsb44JmY&feature=youtu.be or as a Supplementary Material on the editor site of this paper.

## 5. Discussion

Human performance in locomotion tasks is still unmatched by humanoid robots. Because of the lack of assessment methods shared and accepted by the entire robotics community, it is even difficult to estimate the level of maturity of existing technologies. A response to these evaluation needs should induce significant advances in robotics-research. Such an influence of evaluation on the progression of technology performance has been observed in the past, in particular for computer vision and NLP tasks (Martin, 2004).

The definition of evaluation protocols including testing scenarios, testing environments and KPIs or metrics is crucial for the definition of common standards for:

- certifying humanoid robots (i.e., to guarantee the conformity of the product to fixed quality and performance requirements);
- allowing the user to make an informed choice when selecting a specific robot among existing technologies;
- establishing a shared references on which developers and buyers of these technologies can agree in order to define specifications.

This study contributes to the definition of these performance evaluation standards by proposing reproducible experiments and evaluate repeatable performance measurements. These evaluation methods are intended to be passed on to the robotics research community and to standardization committees. In addition we proposed one of the first thorough evaluation of such performance indicators on a human size humanoid robot.

### 5.1. Summary and major outcomes

In this paper we presented a benchmarking for the control architecture described in Figure 1 that was implemented on the HRP-2 robot owned by LAAS-CNRS. The performance indicator used in this paper are mostly based on Torricelli et al. (2015). Based on this work we computed the following set of KPI:

- walked distance,
- success rate,
- maximum tracking error,
- duration of the experiment,
- mechanical joint energy,
- actuators energy,
- cost of transport,
- mechanical cost of transport,
- Froude number.

These KPI represent either the particular characteristics of the experiments or the performances of the control architecture used. The list of algorithms executed on the HRP-2 robot were:

- a flat ground CDPG from Kajita et al. (2003a),
- an analytical flat ground CDPG from Morisawa et al. (2007),
- a nonlinear flat ground CDPG from Naveau et al. (2017),
- a multi-contact CDPG from Carpentier et al. (2016),
- a MPWBC from Koch et al. (2014),
- a WBC which is the stabilizer from Kajita et al. (2007) and Kajita et al. (2001)
- a WBC that computes the joint position from the end-effector plus center of mass trajectories from Mansard et al. (2009)
- a WBC that computes the joint acceleration from the end-effector plus center of mass trajectories used in Carpentier et al. (2016).

The list of environmental conditions where the tests could successfully be performed is:

- a temperature controlled room which provided from 10°C to 35°C,
- a sloped ground of various inclinations ([1°−3.0°]),
- a controlled mobile platform that simulates a translating ground,
- a set of calibrated weight from 5 to 15 kgs,
- a stick equipped with a force sensor at its tip to apply to measure perturbation on the robot,
- different floors with different frictions.

The list of motion performed in the environmental conditions :

- climbing up 10 cm high stairs without handrail,
- climbing up 15 cm high stairs with handrail,
- walking over stepping stones,
- walking on a beam,
- walking on a flat ground,
- walking on a slope,
- walking over obstacles.

From all these results and experiments few major results come out. First the temperature plays a role on the energy consumed during a motion. We observed that the colder the room is, the more mechanical and electrical energy is consumed. We also noticed that the more the motion is at the limit of stability the more the stabilizer has to inject energy into the system to compensate for potential drift. This creates a noticeable increase in energy consumption, e.g., when the robot walks on a beam, steps over obstacle, walks on stepping stones. However the most expensive motion is climbing stairs which is clearly a challenge for future potential applications in which stairs are involved. Finally, in terms of cost of transport, the algorithm proposed by Carpentier et al. (2016) seems to be the most efficient and the most versatile. Its main disadvantage during this campaign was the lack of on-line implementation compared to Morisawa et al. (2007) and Naveau et al. (2017).

### 5.2. Limits

The main limit in the approach proposed here is the difficulty to make the experiments to be more statistically significant. In its current form at least 3 people are needed to perform one experiment, which makes them error-prone and time consuming. Given the wide range of motions that a humanoid robot is able to perform, wear testing needs humanoid robots to be able to fall down and stand up again and restart their behavior. This is a current hot topic in humanoid robotics. The Atlas humanoid robot built by Boston Dynamics has recently demonstrated its capabilities to fall down without breaking and stand up. HRP-2 is an electric-based humanoid robot which is mechanically fragile due to its harmonic drive. Although several works (Fujiwara et al., 2006; Samy and Kheddar, 2015) have developed new approaches toward making such robot more resilient to falling, it is still difficult to implement them in practice due to the cost of failure. In the meantime, benchmarking will help to understand the repeatability and the robustness of the various algorithms implemented on humanoid robots. For very unstructured environments more tests will probably be needed, and a way to classify the environments necessary (using gravels, stairs, size of stairs, different shapes of stairs, or database of environments, forests). But so far such environments can be handled only by a small number of humanoids and the approach proposed in this paper is feasible for a large set of current humanoid robots.

### 5.3. Future work

We could not properly compute the KPI when trying to vary the friction of the ground. A future work is then to implement a proper slip observer like the one in Kaneko et al. (2005). based on this observer, Based on this we should build a stabilizer that could be used in multi-contact motions in order to compensate for external perturbations and modeling assumption. Furthermore, the LAAS-CNRS has acquired a new humanoid robot Talos (Stasse et al., 2017). The future work consists in implementing all the algorithms presented in this paper and perform the benchmarking on this new robot.

## Data availability statement

As a reminder, a CAD model of the staircase used is available on the github repository where all the log of the experiments are also present: https://github.com/laas/koroibot_KPI. All the computation performed on the logs and implementing the key performance indicators are available here: https://github.com/laas/EnergyComputation.

## Author contributions

OS, EB, and KG--E conducted the experiments on the temperature, climbing stairs, at the LNE. MN and OS conducted the experiments with Koroibot. OS, EB, and PS conceived the research idea. PS obtained funding for the project. OS, KG--E, MN, EB, RR, GA, and PS participated in the preparation of the manuscript.

### Conflict of interest statement

The authors declare that the research was conducted in the absence of any commercial or financial relationships that could be construed as a potential conflict of interest. The reviewer FA and handling Editor declared their shared affiliation.
